# The Possibility of Using Fruit-Bearing Plants of Temperate Climate in the Treatment and Prevention of Diabetes

**DOI:** 10.3390/life13091795

**Published:** 2023-08-23

**Authors:** Grzegorz P. Łysiak, Iwona Szot

**Affiliations:** 1 Department of Ornamental Plants, Dendrology and Pomology, Faculty of Horticulture and Landscape Architecture, University of Life Sciences, Dąbrowskiego 159, 60-594 Poznań, Poland; glysiak@up.poznan.pl; 2 Subdepartment of Pomology, Nursery and Enology, Institute of Horticulture Production, Faculty of Horticulture and Landscape Architecture, University of Life Sciences in Lublin, Głęboka 28, 20-612 Lublin, Poland

**Keywords:** diabetes mellitus, antidiabetic properties, temperate climate plants, Cornelian cherry, mulberry, bird cherry, saskatoon, mountain ash, hawthorn, quince, *Vaccinium*, wild roses

## Abstract

Diabetes mellitus is one of the most dangerous metabolic diseases. The incidence of this disease continues to increase and is often associated with severe complications. Plants and natural plant products with a healing effect have been successfully used in the treatment of many disease entities since the beginning of the history of herbalism and medicine. At present, great emphasis is placed on the biodiversity of crops and the replacement of the monoculture production system of popular temperate climate plants, such as apple, pear, plum, and vine, with alternative fruit species. Very promising fruit plants are Cornelian cherry (*Cornus mas*); mulberry (*Morus alba*); bird cherry (*Prunus padus*); sour cherry (*Prunus cerasus*); plants of the genus *Amelanchier, Sorbus,* and *Crategus*; medlar (*Mespilus germanica*); quince (*Cydonia oblonga*); plants of the genus *Vaccinium*; and wild roses. When promoting the cultivation of alternative fruit-bearing plants, it is worth emphasizing their beneficial effects on health. This systematic review indicates that the antidiabetic effect of various parts of fruit plants is attributed to the presence of polyphenols, especially anthocyanins, which have different mechanisms of antidiabetic action and can be used in the treatment of diabetes and various complications associated with this disease.

## 1. Introduction

Diabetes mellitus (DM) is a multifactorial metabolic disease of multifactorial etiology (type 1, 2, and gestational diabetes), characterized by chronic hyperglycemia and disorders of carbohydrate, lipid, and protein metabolism, resulting from inadequate insulin release or insufficient effect of its action [1].

The prolonged period of hyperglycemia first causes damage to blood vessels, resulting in various complications associated with the ischemia of organs such as the heart, brain, kidneys, retina, or lower limbs. Currently, the most common treatments of diabetes are still oral hypoglycemic drugs and insulin therapy. In the pharmacological treatment of diabetes, insulin, sulfonylurea derivatives, glinides, biguanide derivatives, α-glucosidase inhibitors, and glitazones are used [2]. However, the use of drugs often has side effects, for example, the use of biguanide derivatives may cause intolerance of the digestive system manifested by lack of appetite, nausea, vomiting, abdominal pain, and diarrhea. Some patients experience serious metabolic complications, i.e., an increase in the concentration of lactic acid in the blood, which can lead to metabolic acidosis, diabetic coma, and even death. Glitazones, in turn, may cause liver damage, and, therefore, their intake requires the monitoring of serum alanine transferase activity [3]. Due to the many side effects of conventional drug therapy, herbal remedies are sought to support the treatment of diabetes. Currently, in the treatment of diabetes, great emphasis is placed on a balanced diet and lifestyle of patients, which can effectively delay the introduction of drugs [4]. There is a lot of research on products of natural origin that support the treatment of diabetes. For hundreds of years, plant raw materials have been used as basic antidiabetic drugs. Currently, advances in analytical technologies have allowed phytocompounds with antidiabetic properties, such as flavonoids, terpenes, saponins, carotenoids, alkaloids, and glycosides, to be determined in many plants [5]. Hundreds of plants with potential properties supporting the treatment and prevention of diabetes have been identified [6,7]. Very promising plants with antidiabetic properties are those growing in the temperate climate zone; the herbal raw material of those plants is not only fruit, but also leaves, flowers, bark, or roots. Temperate fruit refers to all fruit that typically grows more than 23.5° from the equator, but also includes avocados and citrus fruits, often classified as subtropical. Temperate fruits account for the largest share in fruit production in the world and determine the fruit diet [8]. At present, great emphasis is placed on the biodiversity of crops and the replacement of monoculture production system of popular temperate climate plants, such as apple, pear, plum, and vine, with alternative fruit species. These include Cornelian cherry; white mulberry; bird cherry; cherry; plants of the genus *Amelanchier, Sorbus,* and *Crategus*; medlar *Mespilus germanica*; common quince; plants of the genus *Vaccinium*; and wild roses. The fruit of these plants is available in fresh, dried, frozen, and processed form as juices, jams, and concentrates. In combination with other products, it is used to make syrups, cakes, tarts, and beverages. The leaves of these plants are used to make infusions. Research on cultivated fruit plants places strong emphasis on the health-promoting properties of the fruit and other parts of those plants, because this allows for a wider use of raw materials obtained from them [9,10,11,12,13,14,15,16,17,18,19,20]. When promoting the cultivation of alternative fruit-bearing plants, it is worth emphasizing their beneficial effects on health. The International Diabetes Federation estimated that there were approximately 463 million adults with diabetes in 2019; this figure has been projected to rise up to 578 million by 2030 and to 700 million by 2045 [21]. Considering the alarming rate of diabetes incidence, we have decided to analyze the antidiabetic properties of plants that can increase the biodiversity of commercial crops. Below are discussed alternative plants with edible fruit originating from regions with a temperate climate, parts of which are used in the treatment of diabetes.

## 2. Methods

**Figure 1:**
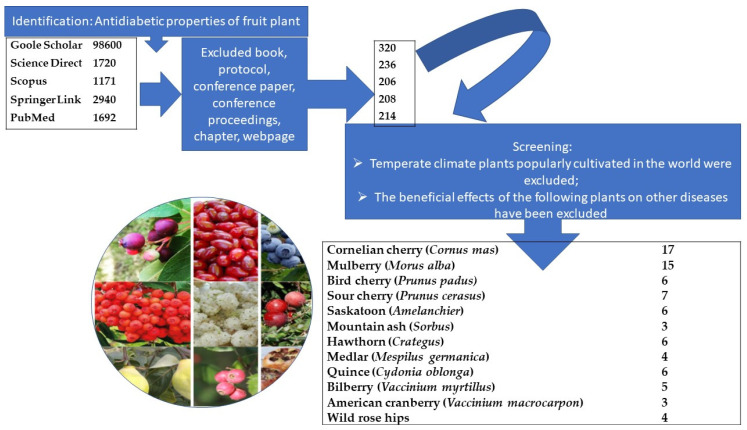
Scheme of the qualitative validation process of the publications used in this review.

A systematic review of the use of fruit plants in the treatment of diabetes was carried out based on scientific publications and patents. The following keyword combinations were used in the search: diabetes mellitus; antidiabetic properties; temperate climate plants, Cornelian cherry; mulberry; bird cherry; saskatoon; mountain ash; hawthorn; quince; *Vaccinium*; wild roses. Searches were carried out using the databases of Google Scholar, Science Direct, Scopus, Springer Link, PubMed, and encompassed the literature since the beginning of the 1990s (the year of the first publication on the subject) to the present. Books, protocols, conference papers, conference proceedings, chapters, webpages were excluded. The focus was on articles published in peer-reviewed scientific journals with high citation rates over the last ten years. The main method was generalization. Popular cultivated fruit plants of temperate climate, whose global annual production exceeds 300,000 tonnes, i.e., apples, pears, peaches, plums, apricots, vines, strawberries, currants, and raspberries, were excluded. Experiments conducted on humans and animals and in vitro, related to the use of selected fruit-bearing plants in reducing diabetes, were analyzed. Attention was paid to which parts of the plant and which substances had a healing effect (Figure 1).

## 3. Cornelian Cherry (*Cornus Mas*)

### 3.1. Characteristics of the Species

Cornelian cherry (*Cornus mas*) is naturally present from central and southern Europe to the Caucasus. Its ease of adaptation to climatic and soil conditions, including high resistance to unfavorable conditions (frost, drought, strong winds), enables it to successfully grow in the temperate climate zone. Cornelian cherry is a long-lived tree or shrub that reaches 3–7 m in height. The fruit is a drupe weighing 1–2 g, with bred cultivars weighing 6–10 g already available. The fruit also varies in shape (oval, spherical, bottle-shaped, pear-shaped) and color (from yellow, through pink, red, carmine, to almost black). Fruit harvest can occur between June and October (depending on cultivar and region) [22]. Ripe drupes fall to the ground; they are moderately juicy, sweetly sweet, and have a sour flavor [23].

Cornelian cherry has been used in herbal medicine for thousands of years. Its use ranges from fighting infections to the treatment of hard-to-heal wounds [24].

### 3.2. Antidiabetic Properties of Fruit (Figure 2a)

Soltani et al. [25] found out that daily consumption of *Cornus mas* L. fruit extract improves glycemic control by increasing insulin levels and reducing serum triglyceride (TG) levels in adult patients with type 2 diabetes. The anthocyanins contained in fruit improve insulin sensitivity through complex biochemical mechanisms, thus potentially preventing diabetes. Furthermore, both animal models of diabetes and cross-sectional studies in humans revealed that anthocyanins reduce blood glucose levels and peripheral insulin resistance [26]. Procyanidins have also been shown to control glucose uptake and translocation of the glucose-4 transporter to the cell membrane in insulin [20]. Anthocyanins can also inhibit the effectiveness of enzymes from the group of hydrolases (glucosidases) that cut the bonds between individual glucose molecules in complex sugars. Glucosidases include, among others, glucosidase, an enzyme that breaks down oligosaccharides and polysaccharides into monosugars in the brush border of the small intestine [8]. Acarbose, migitol, and voglibose are synthetically obtained enzymes that are inhibitors of gastrointestinal glocosidase, resulting in a decrease in postprandial plasma glucose concentration. However, due to side effects such as bloating and abdominal pain, a great deal of attention has been paid to natural inhibitors of these enzymes. Proanthocyanidins can inhibit intestinal glucose absorption. Park et al. [26] reported that Corni Fructus extract can potentially inhibit postprandial hyperglycemia by inhibiting α-glucosidase as one of its antidiabetic effects. Its action was exerted by an active fraction containing polymeric proanthocyanidins. According to these studies, Corni Fructus polymeric proanthocyanidins at a dose of 20 mg per kg of body weight significantly inhibited the increase in blood glucose levels after sucrose load. Inhibition of α-glucosidase via Cornelian cherry extract could be a mechanism for lowering blood glucose levels [10]. Inhibition of α-glucosidase leads to a decrease in the amount of glucose entering the bloodstream. Shishehbor et al. [27], by comparing the effects of water–alcohol extracts of barberry fruit (*Berberis vulgaris*), sour cherry (*Prunus cerasus)* and Cornelian cherry (*C. mas*), showed that the latter had the strongest inhibitory effect on α-glucosidase activity (IC50 6.87 mg·ml^−1^). Dzydzan et al. [28] suggest that the compounds in the *C. mas* fruit probably interfere with the passage, digestion, or absorption of sugars in the small intestine, leading to a decrease in blood glucose levels. Disorders of glucose homeostasis also include abnormalities in lipid metabolism characterized by reduced cholesterol levels in high-density lipoproteins and increased levels of triglycerides, total cholesterol, and cholesterol in low-density lipoproteins [29]. Hyperlipidemia poses a serious risk of premature development of atherosclerosis and cardiovascular complications.

**Figure 2:**
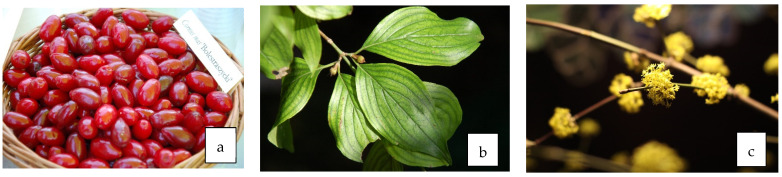
Fruit (**a**), leaves (**b**), and flowers (**c**) of *C. mas* L.

Jayaprakasam et al. [30] observed that a pure mix of the anthocyanins cyanidin 3-*O*-galactoside, pelargonidin 3-*O*-galactoside, and delphinidin 3-*O*-galactoside, as well as ursolic acid present in *C. mas*, had beneficial effects on weight loss, insulin resistance, glucose tolerance, islet function, islet morphology, liver triglycerides, and cholesterol levels in C57BL/6 mice fed a high-fat diet. In another study, Jayaprakasam et al. [31] compared the effects of anthocyanins and anthocyanidins present in Cornelian cherry fruit on the stimulation of insulin secretion by rodent pancreatic cells. They showed that among the four anthocyanins identified in the fruit, cyanidine 3-*O*-glucoside and delphinidin 3-*O*-glucoside were the most effective agents to increase insulin secretion. The anthocyanin found in the largest amounts was pelargonidine 3-*O*-galactoside, and its aglycone–pelargonidine caused a 1.4-fold increase in insulin secretion at a glucose level of 4 mM.

Narimani-Rad et al. [32] determined serum glucose and insulin levels in rats after injecting intraperitoneally (peripherally) Cornelian cherry fruit extract at doses of 50, 100, 200, and 400 mg·kg^−1^ body weight (b.w.). Even the lowest dose significantly reduced the level of glucose, whereas no increase in insulin levels was recorded.

Świerszczewska et al. [33] investigated the possibility of using anthocyanins from cornelian cherry fruit to reduce diseases associated with hyperlipidemia. The most effective fraction that limited the action of the digestive hormone: pancreatic lipase was the one containing pelargonidin 3-*O*-galactoside. The inhibition of digestive enzymes involved in the breakdown of fats and starch is a mechanism for the treatment of obesity and related diseases by reducing fat absorption and the postprandial increase in blood glucose.

People with type 2 diabetes are at risk of complications such as diabetic bone disease. Omelka et al. [29] investigated the effect of cornelian cherry pulp on bone quality parameters in rats with type 2 diabetes. They confirmed the hypolipemic effect of the fruit and also pointed to its protective effect on the bones. A dose of cornelian cherry pulp of 1000 mg·kg^−1^ b.w. had a positive effect on femoral weight, cortical bone thickness, relative volume of trabecular bone, and trabecular thickness. They found that anthocyanins present in fruit prevent oxidative stress which is the root cause of bone damage and reduced strength in chronic hyperglycemia associated with type 2 diabetes.

The intestinal microbiota can affect body weight, insulin sensitivity, sugar, and lipid metabolism, which is why it has been hypothesized [34] that its changes may contribute to the pathogenesis of obesity and diabetes. Particularly important for the proper functioning of the intestinal microflora is the presence of bacteria with health-promoting properties, mainly those belonging to the types *Lactobacillus* and *Bifidobacterium*, and the appropriate supply of prebiotic substances that ensure their proper growth. A critical aspect resulting from the composition of the intestinal microbiota is the pH of the environment in which they occur. Sip et al. [35] found that Cornelian cherry fruit extract, in combination with inulin as a probiotic carrier, favorably modifies intestinal microflora and can contribute to the reduction of the above-mentioned diseases.

### 3.3. Antidiabetic Properties of Leaves (Figure 2b)

Cornelian cherry leaf infusions are traditionally used by the Turkish population to treat hyperglycemia, hypertension, and difficult-to-heal wounds [36]. Hyperglycemia in patients with diabetes induces many molecular changes, changing cellular metabolism and disturbing the oxidative–antioxidant balance. The authors unanimously confirm the increase in free radical production and the intensification of oxidative processes in the course of diabetes. In vivo and in vitro studies by Celep et al. [37] determined the activity of methanol extract from the leaves of *C. mas*. In vitro screening tests showed high antioxidant activity in terms of free radical capture and metal reduction activity. Studies on rats showed that Cornelian cherry leaf extract restored the activity of antioxidant enzymes, reduced the level of lipid peroxidation, and increased the total antioxidant capacity of both blood and liver homogenates of animals. The therapeutic importance was attributed to the presence of gallic acid, a strong antioxidant, in the leaves.

### 3.4. Antidiabetic Properties of Flowers (Figure 2c)

Aldose reductase reduces glucose under hyperglycemic conditions to osmotically active sorbitol, which accumulates intracellularly and may contribute to the development of chronic diabetes complications such as cataracts, neuropathy, and nephropathy. A high concentration of glucose causes the accumulation of sorbitol in cells, which leads to the development of microvascular complications, including retinopathy. Due to their high phenolic acid and flavonoid content, Cornelian cherry flowers may be a potential therapeutic agent in chronic complications of diabetes, as they weaken the effect of aldehyde reductase. Forman et al. [38] determined the ability of *C. mas* flower infusions to inhibit aldose reductase in vitro. They found that the activity of the compounds in the flower infusion was close to the IC50 values of pure plant metabolites, for example, chlorogenic acid, gallic acid, and quercetin, used as references for plant aldose reductase inhibitors.

In sum, anthocyanins, proathocyanins and ursolic acid contained in Cornelian cherry fruit; gallic acid in leaves; and chlorogenic acid, gallic acid, and quercitin in flowers show various mechanisms of antidiabetic activity. This has been confirmed in human studies, but requires further experiments to clarify the method of extraction of plant preparations and their mechanism of action on the human body.

## 4. Mulberry (*Morus*)

### 4.1. Characteristics of the Species

The trees of the genus *Mulberry (Morus*) include 67 species found practically all over the world. Mulberry came to many countries with the development of the silk industry. This was its main use until its medicinal properties were discovered. Herbal material includes mulberry fruit, leaves, flowers, shoots, and bark. The single items of fruit fuse to form fleshy infructescence [39].

### 4.2. Antidiabetic Properties of Fruit (Figure 3a)

Lim et al. [40] studied *M. alba* fruit using 3T3-L1 adipocytes to find potential natural products to help treat type 2 diabetes. They found that rutin and quercetin-3-*O*-β-d-glucoside isolated from fruit improved glucose uptake through the insulin signaling pathway. They influenced body energy homeostasis through enzyme-protein kinase, activated by AMPK-activated protein kinase, which acts as an integrator of regulatory signals monitoring the systemic and cellular energy state of the body. Rutin and quercetin-3-*O*-β-d-glucoside also showed a positive effect on lipid accumulation in adipocytes, suggesting that glucose uptake occurs by activating the AMPK signaling pathway without inducing adipogenesis. The fruit of *M. alba* may be a potential therapeutic candidate for the treatment of type 2 diabetes, without causing side effects such as weight gain.

**Figure 3:**
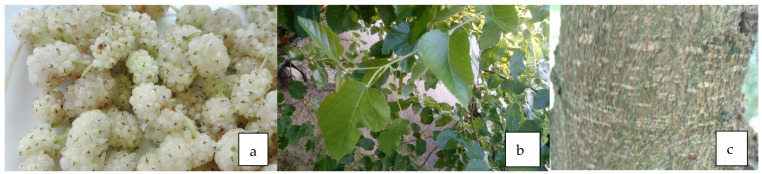
Fruit (**a**), leaves (**b**), and bark (**c**) of *M. alba* L.

Sarikaphuti et al. [41] evaluated the effect of anthocyanin extract of mulberry fruit (*M. alba*) in rats with fatty diabetes with leptin receptor deficiency. They concluded that the anthocyanins present in mulberry fruit—cyanidin 3-*O*-glucoside, cyanidin 3-*O*-rutinoside, pelargonidin 3-*O*-glucoside, pelargonidin 3-*O*-rutinoside—clearly reduce glucose levels, preventing a progressive decrease in insulin secretion.

### 4.3. Antidiabetic Properties of Leaves (Figure 3b)

According to the study by Swathi et al. [42] on rats with diabetes, *M. alba* leaves, which contain rutin, apigenin, quercetin-3-triglyceride, lupeol, β-sitosterol, moracetin, isoquercitrine, coumarin, essential oils, alkaloids, amino acids, and organic acids, have hypoglycemic properties. In addition, they have a soothing effect on diabetic nephropathy caused by streptozotocin-induced oxidative stress. Hwang et al. [43], in a study on patients with diabetes, proved that white mulberry leaves, thanks to the content of deoxynojirimycin—1,5-dideoxy-1,5-imino-D-sorbitol (DNJ)—act as an inhibitor of α-glucosidase, thereby lowering after-the-meal glycemia. The α-glucosidase inhibitors effectively compensate for insufficient insulin secretion in insulin-dependent diabetes mellitus by binding to the enzyme and interfering with the dissolution and absorption of monosaccharides (i.e., glucose). Park et al. [44] showed that mulberry leaf preparations, due to the content of DNJ, quercetin, and soluble dietary fiber, improved post-meal hyperglycemia and some metabolic dysfunctions in rats. They emphasized that the above-mentioned phytocompounds inhibited the glucose transporter and the action of the enzyme α-glucosidase at the border of the intestinal brush. Studies on mice by Chen et al. [45] proved that the mechanism of the hypoglycemic effect of aqueous leaf extract consisted of an increase in glucose uptake. Hunyadi et al. [46] found out in an animal study that mulberry leaf extract lowered fasting glucose levels, due to the presence of chlorogenic acid and rutin.

People with diabetes often suffer from lipid disorders such as hypertriglyceridemia and hypercholesterolemia. Andallu et al. [47] reported a 16% decrease in triglycerides in patients with type 2 diabetes after treatment with capsules filled with mulberry leaf powder. Wilson and Islam [48] administered *M. alba* leaf tea to rats with streptozotocin-induced diabetes for 4 weeks. They found no beneficial antidiabetic effects, i.e., reduction in polyphagia, excessive thirst, weight, blood glucose levels, glucose intolerance, serum insulin, uric acid levels, and liver parameters. However, the total cholesterol in serum as well as LDL cholesterol and triglycerides, decreased significantly, indicating a potential hypolipidemic effect of mulberry leaf tea.

Diabetes may cause kidney problems, resulting in elevated levels of urea in the blood. In studies by Mohammadi and Naik [49], elevated blood urea levels were restored to control levels after treatment with mulberry leaf extract.

Adipose tissue, especially abdominal tissue, produces many hormonally active substances, called adipokines, which may contribute to the growth of insulin resistance and the development of diabetes. One of the goals of diabetes treatment is to reduce weight and maintain a normal BMI. Leaf extracts of *M. alba* were shown to reduce food intake in healthy rats, in rats with diabetes, and in those treated with streptozotocin. The mechanisms attributable to mulberry leaves in the treatment of obesity are the inhibitory effect on digestive enzymes and differentiation of adipocytes, inhibition of digestion and food intake, and a stimulating effect on energy expenditure and lipid metabolism [50]. Melanin concentrating hormone (MCH1), which is expressed mainly in the lateral hypothalamus and incerta zone of the central nervous system, is reported to play an important role in nutrition and energy metabolism. The results obtained by Oh et al. [51] suggest that long-term treatment of obese mice with *M. alba* leaf extract led to weight reduction due to antagonism to the MCH1 receptor.

### 4.4. Antidiabetic Properties of Bark (Figure 3c)

The bark of *M. alba* has traditionally been used in Chinese medicine under the name “Sangbaipi” to treat inflammation, asthma, and heart disease [52]. Liu et al. [53] were the first to isolate *M. alba* sanggenol O, 2,3-trans-dihydromorin, and cuvanon A with stronger weakening properties of the enzyme α-glucosidase compared to acarbose. Singab et al. [54] showed that the alcoholic extract of the root bark of *M. alba* contains the hydrophobic flavonoids morusin, cyclomorusin, neocyclomorusin, kuwanon E, and 2-arylbenzofuran; moracin M; and two terpenes: betulinic and methyl ursolate. In a study on rats, they showed that injection of the extract protected pancreatic cells from degeneration and reduced lipid peroxidation. Jo et al. [55] showed that it is the stulbenoid mulberroside A, present in the roots of *M. alba,* and the oxyresveratrol obtained from it by enzymatic conversion, that are responsible for the antihyperlipidemic properties.

In sum, mulberry leaves are distinguished by a variety of specific components that have a beneficial antidiabetic effect: rutin, apigenin, quercetin-3-triglyceride, lupeol, β-sitosterol, moracetin, isoquercitrine, coumarin, essential oils, alkaloids, amino acids, and organic acids, deoxynojirimycin: 1,5-dideoxy-1,5-imino-D-sorbitol (DNJ. Bark extracts also have a strong antidiabetic effect thanks to the content of sanggenol O, 2,3-trans-dihydromorin, and cuvanon A, morusin, cyclomorusin, neocyclomorusin, kuvanone E and 2-arylbenzofuran, moracin M, and two terpenes (betulinic and methyl ursolate), stulbenoid mulberroside A, oxyresveratrol. Fruit, due to the content of rutin, quercetin-3-*O*-β-d-glucoside and anthocyanins, may be a potential therapeutic candidate for the treatment of type 2 diabetes. The beneficial effect of preparations from various parts of mulberry is proven by studies also conducted on humans and their wide application in the pharmaceutical industry. Further research is needed to refine the extraction method and elucidate the mechanisms of antidiabetic action.

## 5. Bird cherry (*Prunus Padus*)

### 5.1. Characteristics of the Species

Bird cherry (*Prunus padus* L.) grows naturally in a large part of the world, including Africa, Europe, and East Asia. It prefers fertile and moist soils. Some mountainous areas are home to a dwarf *Prunus padus* subsp. *borealis,* or Cajander, which has larger fruit than the type. Bird cherry grows as a strongly branched shrub or tree up to a height of 15 m. Flowers are white, gathered in approximately 15 cm long clusters, with a strong aroma. Bird cherry fruit is edible, being sweet, very tart, and slightly bitter; it ripens in summer, and when overripe, it quickly falls to the ground [56].

### 5.2. Antidiabetic Properties of Fruit (Figure 4a)

Telichowska et al. [57] evaluated the ability of fruit extracts, obtained by various methods, to inhibit the activity of the enzyme α-glucosidase. The most effective was an aqueous solution containing ferulic acid, gallic acid, quercetin, and catechins, at which the activity of α-glucosidase was at the level of 27.11 I50 mg·mL^−1^. The importance of the extraction method was also emphasized by Wen et al. [58], who proved that the ethanolic solution of *Murrayae exotica* leaves, after purification, had a stronger ability to inhibit the activity of α-glycosidase and α-amylase. Plant substances are interesting in terms of antidiabetic efficacy because they can reduce the doses of synthetic antidiabetic drugs. Oboh et al. [59] proved the possibility to reduce the dose of Acarbose™ by administering the drug in combination with gallic acid. According to the authors, the use of a Acarbose™ combined with gallic acid (1:1) in antidiabetic therapy may help reduce the side effects of acarbose.

**Figure 4:**
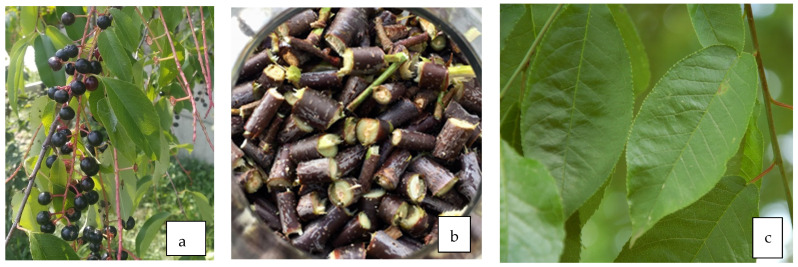
Fruit (**a**), bark (**b**), and leaves (**c**) of *P. padus* L.

### 5.3. Antidiabetic Properties of Bark (Figure 4b)

The chronic production of reactive oxygen species (ROS) by mitochondria can contribute to the development of insulin resistance. Oxidative imbalances in cells are a factor that increases susceptibility to the development of inflammation and disease. Telichowska et al. [60] compared different methods to obtain bird cherry bark extract and found that the acetone–water extract had the highest chelating activity (44.87%), and the ethanolic bark extract showed the highest reducing power. Bark extracts had a stronger antioxidant effect than fruit. Similarly, Korean studies have confirmed the beneficial antidiabetic effects of methanolic extracts of the leaves and branches of *P. padus*. Branch extract was found to show greater activity compared to leaf extract, as a result of the higher content of polyphenols.

### 5.4. Antidiabetic Properties of Leaves (Figure 4c)

Kharyal and Puri [61] detected corosolic acid (8.03 μg·20 mg^−1^ dm) in the leaves of *P. padus*, which was responsible for the strong antidiabetic effect. In the experiment on inhibition of the enzyme glucosidase, Acarbose™ and leaf extract were found to have IC50 values of 193.62 and 114.72 αμg·ml^−1^, respectively. The leaf extract also had a higher ability to capture DPPH free radicals than standard ascorbic acid. Hyun et al. [62], who compared the oxidative activity of leaves, flowers, fruit, and young twigs of *P. padus,* found that the highest values were obtained for water and methanol extracts from leaves. The presence of rutoside, hyperoside, chlorogenic, and caffeic acid was confirmed by the analysis of raw materials.

In sum, the ferulic acid, gallic acid, quercetin, and catechins contained in bird cherry fruit inhibit the activity of the enzyme α-glucosidase, while the compounds found in bark and leaves, such as rutoside, hyperoside, chlorogenic, and caffeic acid, protect against the chronic production of reactive oxygen species that may stimulate the development of insulin resistance.

## 6. Sour Cherry (*Prunus Ceresus*)

### 6.1. Characteristics of the Species

Cherry (*Prunus cerasus* L.) was derived from the crossing of bird cherry (*Prunus avium* L.) with dwarf cherry (*Prunus fruticosa Pall*.). In the wild, it is found in Asia Minor and in almost throughout Europe. It is a shrub or tree that grows to a height of about 10 m, with a dense crown usually without a leader. Cherry fruit is of flattened spherical shape, with a diameter of 1 to 2 cm. The skin color ranges from bright red to almost black. Red flesh varies in terms of taste intensity: it can be sweet or sour-sweet, often tart, and bitter [63].

### 6.2. Antidiabetic Properties of Fruit (Figure 5)

Cherry fruit is rich in anthocyanins. These natural dyes of plants that accumulate as the fruit ripens are more abundant in ripe fruit. Thus, the color of the fruit is a good indicator not only of ripeness, but also of anthocyanin content [64]. Anthocyanins have properties that stimulate insulin release in β-cells of the pancreas in vitro. The anthocyanins and quercetin contained in cherries have a protective function against obesity, reducing insulin resistance and preventing the onset of diabetes. The bioactive compounds in cherries prevent the risk of diabetes by increasing insulin secretion and metabolism. In studies, 3-*O*-β-d-glucoside has shown glucose-lowering effects in animal model studies. Ataie-Jafari et al. [65] investigated whether concentrated cherry juice favorably alters serum glucose levels in people with type 2 diabetes. After six weeks, a significant reduction in body weight, in total cholesterol, and LDL was observed in patients with diabetes mellitus and hyperlipidemia. Papp et al. [66] in a study on rats also observed a beneficial effect of a diet enriched with freeze-dried cherry fruit: it lowered total and LDL cholesterol and increased the level of HDL cholesterol in the serum of animals with hyperlipidemia. Th study showed that cherries with higher content of polyphenolics had a more pronounced effect on the proper level of cholesterol in the blood, suggesting that in addition to anthocyanins, colorless polyphenols also have a lipid-lowering effect. Van Der Werf [67], showed in a study on rats that cherry consumption lowers the risk of developing diabetes disorders by reducing the accumulation of fat, body weight, and lipid concentration, and improving glucose and insulin regulation and the metabolic and oxidative balance in plasma.

**Figure 5:**
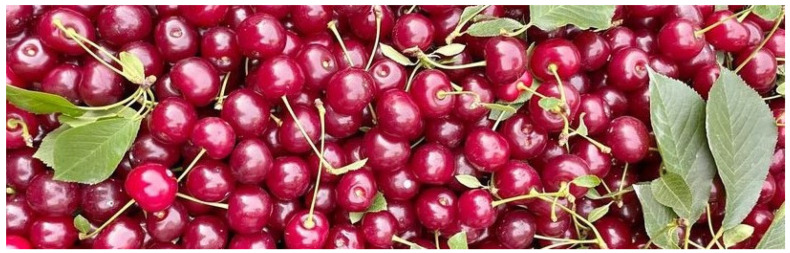
Fruit of *P. cerasus* L.

Complications in diabetes are associated with oxidative stress. Šarić et al. [68] evaluated in vivo the antioxidant efficacy (superoxide dismutase, SOD; catalase, CAT; glutathione peroxidase; Gpx), lipid peroxidation (LPO), and anti-inflammatory properties (cyclooxygenase-2; COX-2) of juices made from the fruit of an autochthonous cherry variety (*Prunus cerasus* cv. Maraska) cultivated in Croatia. They showed that cherry juice has antioxidant effects due to increased SOD (in the liver, blood) and Gpx (liver) and decreased LPO. The study highlights that cherry juice is a potent inhibitor of COX-2 and an antioxidant in the liver and blood of mice, but no such effect was reported in the brain. Wojdłyo et al. [69] proved a strong correlation between the antioxidant properties of cherry fruit and the content of polymeric procyanidins. Goncalves et al. [70] observed that the antioxidant activity of cherry is strongly correlated with the content of p-coumaroylquinic acid and also tends to correlate with the concentration of catechin monomer. Piccolella et al. [71] found that flavonoids and quinic acid are the substances with the highest antioxidant properties among the bioactive components of cherries. In a study by Van Der Werf et al. [67], a diet enriched with cherries helped maintain antioxidant and anti-inflammatory status, leading to a reduction in vascular and liver complications.

In sum, compounds contained in sour cherry fruit, such as anthocyanins and colorless polyphenols, favorably alter serum glucose levels, positively affect the blood lipid profile, and improve oxidative balance in plasma. However, more human experiments are needed.

## 7. Plants of the Genera *Amelanchier*

### 7.1. Characteristics of the Species

This genus contains about 25 wild and cultivated species, which are often difficult to distinguish due to the high variability of foliage. Serviceberry (*Amelanchier ovalis*) grows in Europe, whereas the two most popular species in North America are saskatoon berry (*Amelanchier alnifolia*) and *Amelanchier lamarckii* (syn. *A. canadensis* Koch), which, in addition to the decorative qualities of trees, have tasty fruit that can be eaten fresh and is also a valuable raw material for processing. Species that occur in America and can be used in processing or pharmacy also include *Amelanchier laevis* Wiegand, with the largest (in diameter) and tastiest fruit (18 mm); and *Amelanchier florida* Wiegand, with purple fruit with a diameter of 10–13 mm [72].

### 7.2. Antidiabetic Properties of Fruit (Figure 6a)

The bioactive ingredients contained in Saskatoon berries, especially polyphenols, can lower blood glucose levels and regulate glycogen accumulation. For this reason, they may be useful in the treatment and prevention of diabetes [73].

**Figure 6:**
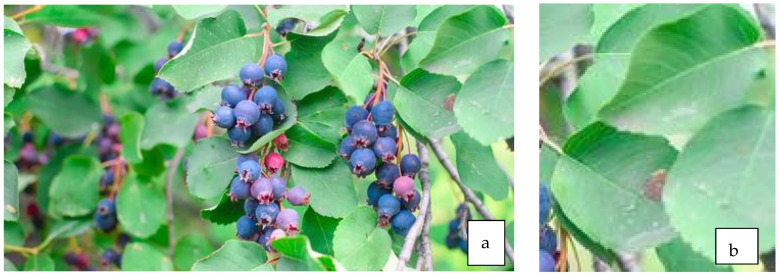
Fruit (**a**) and leaves (**b**) of *A. alnifolia*.

Juríková et al. [74] determined the flavonoid profile of saskatoon fruit (*Amelanchier alnifolia* Nutt.) and their health-promoting properties. They showed that the fruit abounds in flavonoids, among which the main classes are flavonols (quercetin and rutin), flavans (proanthocyanidin compounds from dimers to heptomers and even higher polymers), and finally, anthocyanins. Kraft et al. [75] studied the fruit of four wild species, including *Amelanchier alnifolia*, for the health-promoting properties of their phytochemicals. They performed a number of biological tests that evaluated the potential effect of fruit on the following microvascular complications of diabetes: hyperglycemia, expression of pro-inflammatory genes, and symptoms of metabolic syndrome. Nonpolar components (carotenoids) found in the fruit of *Prunus virginiana*, *Shepherdia argentea*, and *Viburnum trilobum* showed strong inhibition of aldose reductase. Polar components, in turn, mainly phenolic acids, anthocyanins, and proanthocyanidins, had a hypoglycemic effect and inhibited the expression of genes, which are anti-inflammatory markers of interleukin 6 (IL-6) and cyclooxygenase-2 (COX-2) [76]. They also confirmed that the anthocyanin and proanthocyanidin fraction found in the fruit has a hypoglycemic effect and inhibits the expression of the IL and COX-2 genes. The fruit of related species, *A*. *canadensis* and *A. arborea*, also inhibited cyclooxygenase-1 and -2 in vitro, indicating a role in alleviating inflammation [77].

### 7.3. Antidiabetic Properties of Leaves (Figure 6b)

Zhang et al. [78] investigated the effectiveness of an aqueous extract of *A. alnifolia* leaves in inhibiting α-glucosidase activity in mice with diet-induced hyperglycemia. The saskatoon leaf extract showed a significant inhibition of α-intestinal glucosidase and delay in carbohydrate absorption, resulting in a decrease in postprandial blood glucose, similar to the antidiabetic drug Acarbose™.

Patients with diabetes are exposed to vascular complications. In hyperglycemia conditions under the influence of increased glucose metabolism in endothelial cells, granulocytes, monocytes, and platelets, there is an increased production of free oxygen radicals [79]. Meczarska et al. [80] studied structural changes in the membrane of red blood cells under the influence of saskatoon fruit and leaf extracts. The cell membrane is a protective barrier against substances harmful to the body, particularly free radicals. The extracts are a rich source of polyphenols, mainly flavonoids, with flavonols dominating in the leaves and anthocyanins in the fruit. The extracts modified the physical properties of the erythrocyte membrane to varying degrees, causing the formation of echinocytes, an increase in osmotic resistance, and changes in the polar part of the membrane. The polyphenolic compounds contained in the saskatoon extracts did not destroy the biological membrane and effectively protected it against oxidation by interacting with the membrane surface.

In sum, polar components of saskatoon fruit, mainly flavonoids such as phenolic acids, anthocyanins, and proanthocyanidins, have a hypoglycemic effect, while leaves rich in flavonols protect cells against oxidative stress. Further studies on humans are necessary.

## 8. Plants of Genera *Sorbus*

### 8.1. Characteristics of the Species

The genus *Sorbus* includes 250 species of trees and shrubs native to East Asia and distributed in temperate climates in the Northern Hemisphere. Some of the species grow in the far north of Siberia and Norway. The most important of them is mountain ash (*Sorbus aucuparia* L.). It grows almost throughout Europe, western Siberia, and Asia Minor, both in the lowlands and in the mountains. Above the upper border of the forest, it generally takes on a bushy form. When growing in optimal conditions, it is a small tree up to 15 m in height. The fruit is fleshy spherical berries, scarlet red when fully ripe, with a bitter and tart taste. It ripens in autumn and persists on the tree long after the leaves fall. The genus *Sorbus* also includes white beam (*Sorbus aria* L.), wild service tree (*Sorbus torminalis* L.), and Swedish mountain ash *(Sorbus intermedia* L.) [81].

### 8.2. Antidiabetic Properties of Fruit (Figure 7a)

Hasbal et al. [82] investigated the antidiabetic activity in vitro of aqueous extracts from fruit of *S. aucuparia* and *S. torminalis* by measuring the inhibition of important digestive enzymes: α-glucosidase and pancreatic α-amylase. They found that the total content of phenols and flavonoids is closely correlated with antidiabetic activity. Extracts of *S. torminalis* and *S. aucuparia* showed strong inhibitory effects of α-glucosidase, while only *S. torminalis* moderately inhibited the activity of pancreatic α-amylase. Grussu et al. [83] investigated in vitro the effect of the fruit extract of *S. acuparia*, which was rich in proanthocyanidins, on the inhibition of α-amylase. The co-incubation of proanthocyanidins from mountain ash with acarbose reduced the concentration of Acarbose™ required for the effective inhibition of α-amylase.

**Figure 7:**
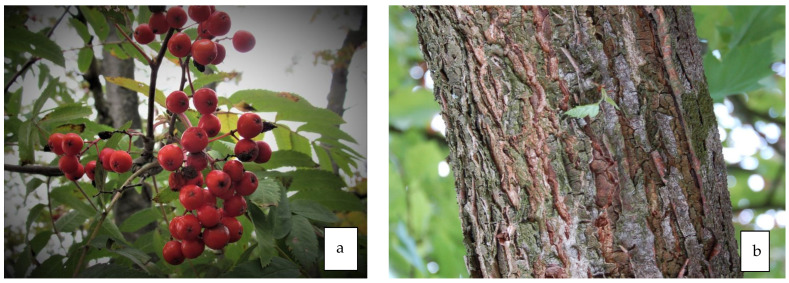
Fruit of *S. acuparia* L (**a**). and bark of *S. decora* (**b**).

### 8.3. Antidiabetic Properties of Bark (Figure 7b)

Vianna et al. [84] evaluated the antidiabetic potential of *S. decora* in in vivo models of insulin resistance and diabetes in rats with type 1 diabetes, in mice with genetic type 2 diabetes and in rats with insulin resistance. The effects of 6 weeks of treatment with the crude ethanol bark extract of *S. decora* were compared with the effects of metformin. *S. decora* extract was shown to have both antihyperglycemic and insulin-sensitizing effects in vivo.

In sum, phenols and flavonoids contained in mountain ash fruit can support the treatment of diabetes with Acarbose™, while bark extract complements the action of metformin. Human studies are recommended.

## 9. Plants of Genera *Crategus*

### 9.1. Characteristics of the Species

This genus includes about 280 easily interbreeding species, which are found mainly in Europe, West Asia, North Africa, and North America. The most popular are *C. monogyna* Jacq., *C. Laevigata*, son. *C oxyacantha* L. These are shrubs or trees up to 5–10 m high. The fruit diameter is up to 1 cm. Single-necked hawthorn has one seed, which has a fragile cover on the outside. The fruit of double-necked hawthorn have 2–3 seeds without such a cover. The fruit is spherical or ovate-spherical with a brownish-red color. It ripens in autumn [85].

### 9.2. Antidiabetic Properties of Fruit (Figure 8a)

In addition to the common complications mentioned above, diabetes may also cause mild cognitive impairment in the elderly. The reason for this is cerebrovascular changes and neurodegeneration, which are exacerbated by diabetes. Pirmoghani et al. [86] investigated the effects of *Crategus* fruit extract, rich in phenols and flavonoids, on blood glucose levels, lipid profile, and memory deficit produced by streptozocin-induced diabetes. They showed that due to its oxidative properties, the Crategus fruit extract had the potential to protect against diabetes and improve the memory of diabetic rats. Similarly, Zarrinkalam et al. [87] found that natural antioxidant bioactive compounds from *Crategus* fruit in combination with training have a beneficial synergistic effect on impaired learning and memory functions in rats with type 1 diabetes. Gao et al. [88] showed that combination therapy with hawthorn fruit extract and metformin helps to reduce body weight, inhibit the expression of the hs-CRP protein, lower liver enzyme levels and fatty liver disease in patients with non-alcoholic fatty liver disease, and prevent diabetic conditions.

**Figure 8:**
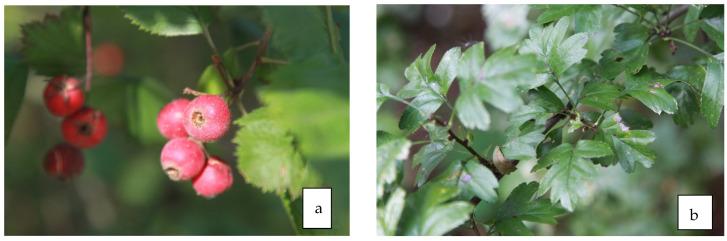
Fruit (**a**) and leaves (**b**) of *C. submollis*.

### 9.3. Antidiabetic Properties of Leaves (Figure 8b)

Diabetic kidney disease is the main microvascular complication of diabetes, whose incidence has increased greatly worldwide. Qin et al. [89] investigated the effects of Crategus leaf flavonoids on damaged kidney tissue in rats caused by oxidative stress. Hawthorn leaf extract improved the general body condition and weight and reduced protein levels in urine. Rats treated with hawthorn leaf extract had significantly lower levels of urea nitrogen, creatinine, triglycerides, and malonic dialdehyde and significantly higher levels of nitric oxide and superoxide dismutase than control rats. Li et al. [90] demonstrated the protective effect of hawthorn leaf flavonoids on fatty liver induced by a high-fat diet. Furthermore, hawthorn leaf flavonoids dramatically increased circulating adiponectin levels and increased adiponectin receptor expression in the liver, indicating a strengthening of the adiponectin pathway in the liver of rats with nonalcoholic fatty liver.

In sum, phenols and flavonoid compounds in hawthorn fruit support through strong antioxidant activity the treatment of diabetes complications such as cognitive impairment in the elderly, while leaf extract protects kidney tissues against antioxidant stress and have a protective effect on fatty liver. However, this requires confirmation in human clinical trials.

## 10. Medlar (Mespilus Germanica)

### 10.1. Characteristics of the Species

Medlar (*Mespilus germanica* L.) occurs naturally in Central Asia, Asia Minor, and the Caucasus. It grows slowly as a shrub or small sapling to a height of 3 to 5 m. It forms a wide, usually low-set crown. It has spherical or pear-shaped fruit, broadly oval at the top, 30 to 60 mm in diameter, with five calyx sepals at the apex, which do not dry out after flowering, but develop with the fruit [91].

### 10.2. Antidiabetic Properties of Fruit (Figure 9a)

Żółnierczyk et al. [92] showed that aqueous and methanol extracts from *M. germanica* fruit inhibited α-amylase activity against acarbose. With dose reduction, the activity of inhibition of α-amylase decreased, with the aqueous extract showing the highest activity compared to other fractions. Katanić Stenkovi et al. [93] compared the antidiabetic properties of *M. germanica*, *Prunus spinosa*, and *Crategus monogyna*. The strongest inhibitory effect of glucosidase expression was shown by fruit extracts of *M. geramanica* and *P. spinosa*, and it was stronger than acarbose. The extract of the *M. germanica* fruit stood out in terms of the content of phenolic compounds as it contained pinocembrin.

**Figure 9:**
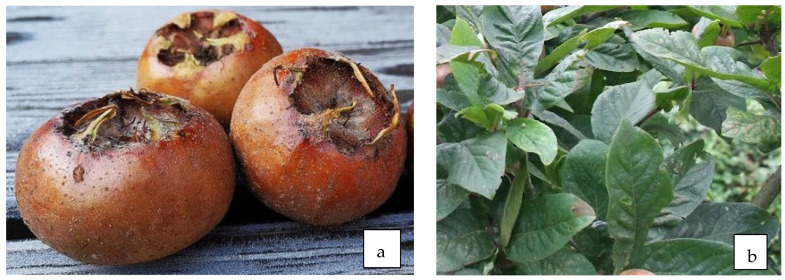
Fruit (**a**) and leaves (**b**) of *M. germanica* L.

### 10.3. Antidiabetic Properties of Leaves (Figure 9b)

Shafiee et al. [94] investigated the effect of *M. germanica* leaf extract in an experiment in mice with streptozotocin-induced diabetes. Oral application of the extract over a 21-day period significantly more reduced glucose levels, oxidative stress, and lipid peroxidation and helped to maintain normal body weight of the animals during the treatment period compared to metformin.

In sum, bioactive compounds from medlar fruit, including pinocembrin, reduce the activity of α-amylase, similarly to acarbose, while the leaf extract lowers glucose levels, oxidative stress, and lipid peroxidation, comparable to metformin. Further studies on the chemical composition of bioactive compounds in fruit and leaves and clinical trials in humans are recommended.

## 11. Quince (Cydonia Oblonga)

### 11.1. Characteristics of the Species

The homeland of quince is the Caucasus, from where it went to Asia Minor, and then to Greece and Rome. Pliny in his books described six cultivars of quince. At that time, quince fried in honey was a very popular desert. Quince is grown in the countries of the Mediterranean and Black Sea basins. From the Iberian Peninsula, quince reached Latin America. From England, it reached Australia and New Zealand [95]. Two forms have been identified within this species: *Cydonia oblonga* subsp. *pyriformis*, with pear-shaped fruit; and *Cydonia oblonga* subsp. *maliformis*, with apple-shaped fruit. Quince usually grows in the form of a tall shrub or tree of 3 to 7 m in height. The fruit of wild quince is quite small, 3–5 cm in diameter, whereas that of cultivated quince varieties is much larger and evenly distributed. The flesh is hard, not very juicy, tart, and aromatic. The parenchyma contains numerous stone cells [96].

### 11.2. Antidiabetic Properties of Fruit (Figure 10a)

Adiponectin is a protein synthesized by adipose tissue and not only has a beneficial effect on glucose and fat metabolism, but also has a protective effect on blood vessels. Lee et al. [97] showed that the aqueous fruit extract of *C. oblonga* was effective in treating obese mice due to the high-fat diet, since it increased adiponectin and HDL cholesterol, while reducing body weight, body fat mass, serum insulin, triglycerides, and leptin levels. Khail et al. [98] studied various extracts from powdered seeds of *C. oblonga.*, Hydroxyl radical and DPPH trapping tests were performed in vitro to determine antioxidant activity. Antidiabetic properties were tested by α-glucosidase and α-amylase tests. Among the extracts tested, the ethanol extract had the best antioxidant and antidiabetic properties. The results indicate that the raw quince seed extracts are an essential source of diabetes treatment. Mirmohammadu et al. [99] investigated the effect of an aqueous extract of *C. oblonga* fruit on the lipid profile of rats with streptozotocin-induced diabetes,. The extract showed a hyperlipidemic effect, as evidenced by a decrease in serum triglycerides, total cholesterol, and LDl, and an increase in HDL levels. In diabetic rats treated with an aqueous solution of *C. oblonga* fruit, serum reductions in liver dysfunction were observed, including alanine tranminase, asparginate transminase, and alkaline phosphatase. Orally administered extract prevented a diabetic-induced increase in serum urea and creatinine, which are biomarkers of kidney dysfunction.

**Figure 10:**
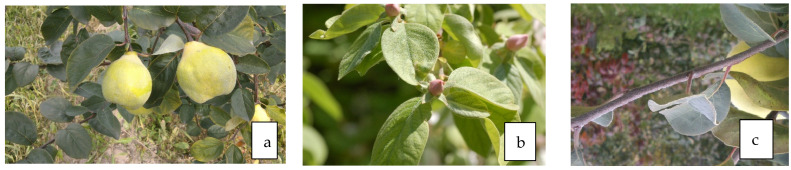
Fruit (**a**), leaves (**b**), and bark (**c**) of *C. oblonga* Mill.

### 11.3. Antidiabetic Properties of Leaves (Figure 10b)

Ferreira et al. [100] investigated that quince leaves at all stages of maturity (calf, yellow, and brown) contained phenols, flavonoids, organic acids, and vitamin E and the minerals Ca, K, Mg, Fe, Cu, Mn, and P, and are therefore a potential source of protection against diseases associated with inflammation, including diabetes. The polyphenol content of quince leaves was higher than that of tea, making them a safe and inexpensive alternative to conventional treatment. Akyurt et al. [101] compared the ability of leaf extracts of *C. oblonga* and six other species: *V. vinifera*, *Morus alba*, *Phaseolus vulgaris*, *Prunus avium*, *Urtica dioica*, and *Beta vulgaris*, to inhibit α-glucosidase activity. Of the examined leaves, the leaves of *V. vinifera*, *C. oblonga*, and *Urtica dioica* showed the strongest inhibition of α-glucosidase.

### 11.4. Antidiabetic Properties of Bark (Figure 10c)

Abed et al. [102] investigated the effectiveness of the methanolic bark extract of *C. oblonga* in inhibiting α-glucosidase and α-amylase activity. They found that due to the richness of phenolic compounds and flavonoids, this extract showed a % inhibition of α-glucosidase and α-amylase (78.21 and 77.98%) comparable to acarbose (87.60 and 85.99%). These extracts also had strong abilities to scavenge free radicals.

In sum, bioactive compounds contained in quince fruit support weight loss and improve the blood lipid profile. The seeds and the bark contain antioxidant compounds and have an inhibitory effect on the activity of α-glucosidase and α-amylase enzymes. On the other hand, phenols, flavonoids, organic acids, vitamin E, and the mineral compounds prevent the inflammatory processes associated with diabetes. This needs to be proven in human studies.

## 12. Plants of Genera *Vaccinium*

### 12.1. Bilberry

#### 12.1.1. Characteristics of the Species

Bilberry (*V. myrtillus*) is a small shrub that grows 35–60 cm tall. It prefers acidic forest soils, mountain mineral heaths, and old peat bogs in the central and northern parts of Europe. The fruit is dark blue or black on the inside and outside and has a diameter of 5–9 mm. It ripens from July to September [103].

#### 12.1.2. Antidiabetic Properties of Fruit (Figure 11a)

De Mello et al. [104] have shown that the consumption of bilberry fruit results in improved cell function and glycemic control in diabetic patients. They found that the consumption of bilberry fruit increases the concentration of hippuric acid in fasting serum, and in the long run, it can improve glucose and insulin metabolism. It turns out that bilberry fruit extracts improve eye microcirculation and lower pressure in the eyeball. Hence, it has importance in both the prevention and supportive treatment of the following eye diseases: twilight amblyopia, cataracts, macular degeneration, diabetic retinopathy [69]. Bilberries have the ability to assume the role of angiotensin-converting enzyme in human angotel cells and have shown their effectiveness in cardiovascular diseases along with antidiabetic properties [105].

**Figure 11:**
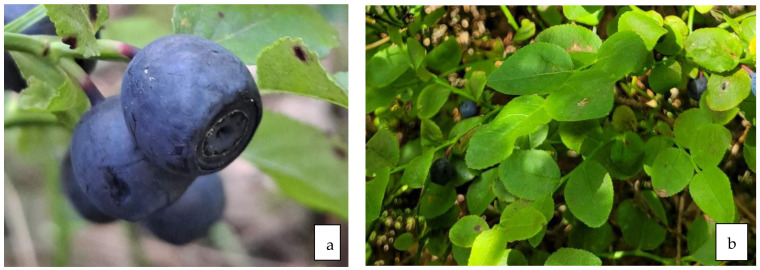
Fruit (**a**) and leaves (**b**) of *V. myrtillus* L.

#### 12.1.3. Antidiabetic Properties of Leaves (Figure 11b)

Bilberry leaves are traditionally used as an auxiliary drug (in conjunction with a proper diet) in the initial stages of type 2 diabetes [106]. In in vitro studies, extracts from leaves of *Vaccinium myrtillus* were found to inhibit the activity of enzymes that break down polysaccharides into simple sugars (α-glucosidase and *α*-amylase). This effect was tested in healthy, obese, prediabetic, and full-blown diabetic rats. In the cited study, inhibition of the increase in blood glucose levels was observed after the administration of a starchy meal [107]. Due to the fact that chlorogenic acid and flavonoids are present in bilberry leaves, it was checked whether the extract of the raw material would improve glucose homeostasis and insulin sensitivity in mice with obesity and diabetes. The study noted positive effects associated with improved insulin sensitivity, inhibition of triglyceride synthesis, and increased use of lipids in the liver and white adipose tissue. Leaf extract prevented hyperglycemia by improving the function of pancreatic beta cells. Body weight was reduced as well [108].

In sum, in animal studies, extracts from bilberry fruit and leaves showed a beneficial effect on the inhibition of enzymes that break down polysaccharides into simple sugars, as well as alleviating the effects of diabetic complications. It is necessary to explain the antidiabetic mechanism of action of specific bioactive substances from bilberry and to confirm it in human studies.

### 12.2. American Cranberry

#### 12.2.1. Characteristics of the Species

The first reports on the cultivation of *Vaccinium macrocarpon* date back to 1816. It was grown in the so-called swamps or rain-flooded meadows in the northern United States. Cranberries produce long creeping shoots (up to 1.5 m long), from which grow short (5–12 cm long), upright, fruit-bearing shoots (5–12 cm long). Its spherical or oval fruit (up to 2 cm in diameter) ripens in autumn and is collected until autumn frosts [109].

#### 12.2.2. Antidiabetic Properties of Fruit (Figure 12)

Cermak et al. [110] showed that quercetin glucosides inhibit glucose uptake into the vesicles of the brush membrane of the pig intestine. Strobel et al. [111] demonstrated that the myricetin present in fruit can inhibit glucose assimilation via the type 4 glucose transporter by rat adipocytes. Schell et al. [112] included patients with type 2 diabetes in a 12-week study to demonstrate that the bioactive components of cranberries help with symptoms of metabolic syndrome and diabetes. A diet enriched with low-calorie cranberries had a significant effect on improving postprandial glucose levels induced by high-fat breakfast and selected biomarkers of inflammation and oxidation in participants with type 2 diabetes. In the study by Rocha et al. [113], daily consumption (240 mL) of cranberry juice and blueberry extract (9.1–9.8 mg of anthocyanins) for 12 weeks for 8 to 12 weeks improved glucose control in patients with type 2 diabetes.

**Figure 12:**
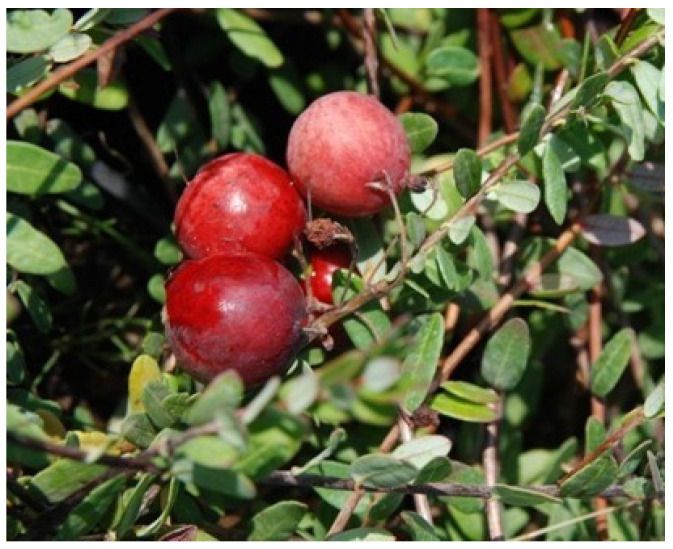
Fruit of American cranberry.

In sum, the promising results of clinical trials on the beneficial antidiabetic effect of extracts of American cranberry fruit–which is now frequently used in preventing urinary tract infections [114]—may contribute to a wider use of this raw material in pharmaceuticals.

## 13. Wild Rose Hips—Plants of Genera *Rosa*

### 13.1. Characteristics of the Species

The name ‘wild rose’ refers to a group of roses that are native throughout the world. Wild roses have five-petaled flowers (or a multiple of five), and their fruit is varied in size and shape. They form shrubs that reach up to 3 m in height. This group includes about 200 species of roses, of which 30 occur in Europe, including, among others, *R. cinnamomea*, *R. pendulina*, *R. canina R. dumalis*, *R. sherardii*, *R. villosa*, *R. tomentosa*, *R. rubiginosa*, *R. agrestis*, *R. indora*, *R. mollis*, *R. jundzillii*, *R. micrantha*, *R. zalana* [115].

### 13.2. Antidiabetic Properties of Fruit (Figure 13a,b)

In a study conducted in mice, Ninomiya et al. [116] observed that acetyl extract of *R.canina* fruit and seeds prevented weight gain in mice fed a normal diet. They explained this by the presence of trans-tyliroside in the extract, which inhibited weight gain and improved glucose tolerance. The effect of trans-tyliroside was even stronger than that of orlistat, a commonly used drug, so this compound can be used to develop a new generation of anti-obesity drugs. In another experiment [117] on mice fed high-fat foods that cause obesity and insulin resistance, it was shown that the addition of rose hip powder was able to prevent—as well as reverse—diet-induced obesity and glucose intolerance. The addition of rose hips reduced lipid levels. Anderason et al. [118] investigated the effects of rose hip drinks taken for 6 weeks by obese patients with normal or impaired glucose tolerance. Significant reductions in systolic blood pressure, total plasma cholesterol, and LDL/HDL ratio were observed. Rose hip drinks were also found to reduce the risk of cardiovascular disease according to the Reynolds risk assessment.

**Figure 13:**
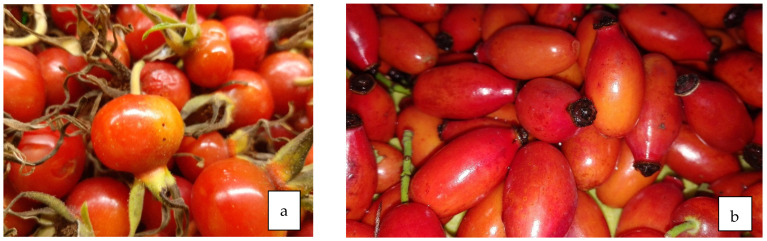
Fruit of *R. rugosa* (**a**) and *R. canina* (**b**).

In sum, studies conducted on animals and humans prove the beneficial antidiabetic effect of rose hip extracts, which can be used in therapy limiting the use of synthetic drugs.

## 14. Conclusions

This review demonstrates that alternative fruit crops of temperate climates deserve attention not only because of their valuable fruit, but because that can increase biodiversity of food. Individual plant raw materials, such as flowers, fruit seeds, leaves, bark, or shoots, derived from fruiting plants, abound in antioxidant and anti-inflammatory bioactive compounds, which improve glucose homeostasis and insulin resistance through various mechanisms of antidiabetic activity. The described plant materials:

- Inhibit the activity of the enzyme α-glucosidase and α-amylase;
- Have a protective and regenerating effect on pancreatic β-cells in the islets of Langerhans, thus increasing insulin secretion;
- Have a hypolipidemic effect by reducing lipid peroxidation and improve dysfunction of the adipocytes responsible for lipid metabolism;
- Alleviate the effects of diabetes complications.

Synergistic interactions of bioactive compounds from plant material can help reduce the amount of synthetic drugs taken in postprandial glycemic control in patients with type 2 diabetes. Individual parts of the described fruit plants have antidiabetic properties, comparable to conventional drugs, and can be consumed at a lower cost. References reporting the effect of those plants in human or human cells are still lacking, in contrast to reports in rats or mice.

## Data Availability

Not applicable.
